# Prediction of ischemic stroke in elderly hypertensive patients using carotid plaque superb microvascular imaging characteristics: a lasso-logistic regression model

**DOI:** 10.1007/s10396-024-01513-0

**Published:** 2025-03-15

**Authors:** Xiuli Jin, Xiangxi Yang, Fengjuan Li

**Affiliations:** Department of Ultrasound Diagnosis, Chengde Central Hospital, Chengde, 067000 Hebei Province China

**Keywords:** Elderly Hypertensive Patients, Carotid Plaque, Ischemic Stroke, Superb Microvascular Imaging (SMI), Logistic Regression Prediction Model

## Abstract

**Purpose:**

This study aims to assess the effectiveness of Superb Microvascular Imaging (SMI) in evaluating intraplaque neovascularization (IPN) in carotid plaques and its association with ischemic stroke in elderly hypertensive patients, and to develop and validate a prediction model for ischemic stroke based on SMI characteristics.

**Methods:**

This retrospective study included 314 elderly hypertensive patients with carotid plaques, divided into a training cohort (235 cases) and a validation cohort (79 cases). Patients were categorized into stroke and non-stroke groups. SMI characteristics of carotid plaques and baseline variables were analyzed using univariate logistic regression and Least Absolute Shrinkage and Selection Operator (LASSO) regression to develop a multivariate logistic regression model. The model was then validated.

**Results:**

In the training cohort, 79 patients (33.6%) experienced ischemic stroke. Significant predictors included BMI, hypertension grade, IPN grade, and stenosis percentage. These factors were incorporated into a logistic regression model, which was validated with an area under the curve (AUC) of 0.79, 69.6% accuracy, 60.8% sensitivity, and 85.7% specificity.

**Conclusion:**

BMI, hypertension grade, IPN grade, and carotid plaque stenosis are associated with ischemic stroke in elderly hypertensive patients. The developed logistic regression model based on these indicators can improve the prediction of ischemic stroke in this population.

**Supplementary Information:**

The online version contains supplementary material available at 10.1007/s10396-024-01513-0.

## Introduction

Ischemic stroke (also known as “cerebral infarction”) occurs with high frequency elderly patients with hypertension, exhibiting significant mortality and morbidity rates [1]. The occurrence of cerebral infarction is closely related to the stability of carotid atherosclerotic plaques [1, 2]. Studies have shown that intraplaque neovascularization (IPN) in the artery plaque is associated with plaque instability that often coexists at multiple sites within the systemic vascular bed [3]. The carotid IPN can predict the incidence of significant coronary artery disease (CAD) [4] and cerebrovascular events [5].

Superb Microvascular Imaging (SMI) technology is highly sensitive to imaging low-speed microvessels (with a lumen diameter > 0.1 mm) and has advantages in identifying plaque vulnerability [6]. Currently, studies have confirmed that SMI can effectively detect carotid plaque neovascularization with accuracy comparable to contrast-enhanced ultrasound, offering a promising noninvasive alternative for assessing plaque stability [7]. However, it has yet to be determined whether carotid SMI characteristics is able to predict future ischemic stroke for the purpose of risk stratification. This study aims to explore the diagnostic value of SMI technology in assessing intraplaque neovascularization within carotid plaques and seeks to establish a clinical prediction model for ischemic stroke based on carotid plaque SMI characteristics.

## Materials and methods

### Cohort

**Fig. 1:**
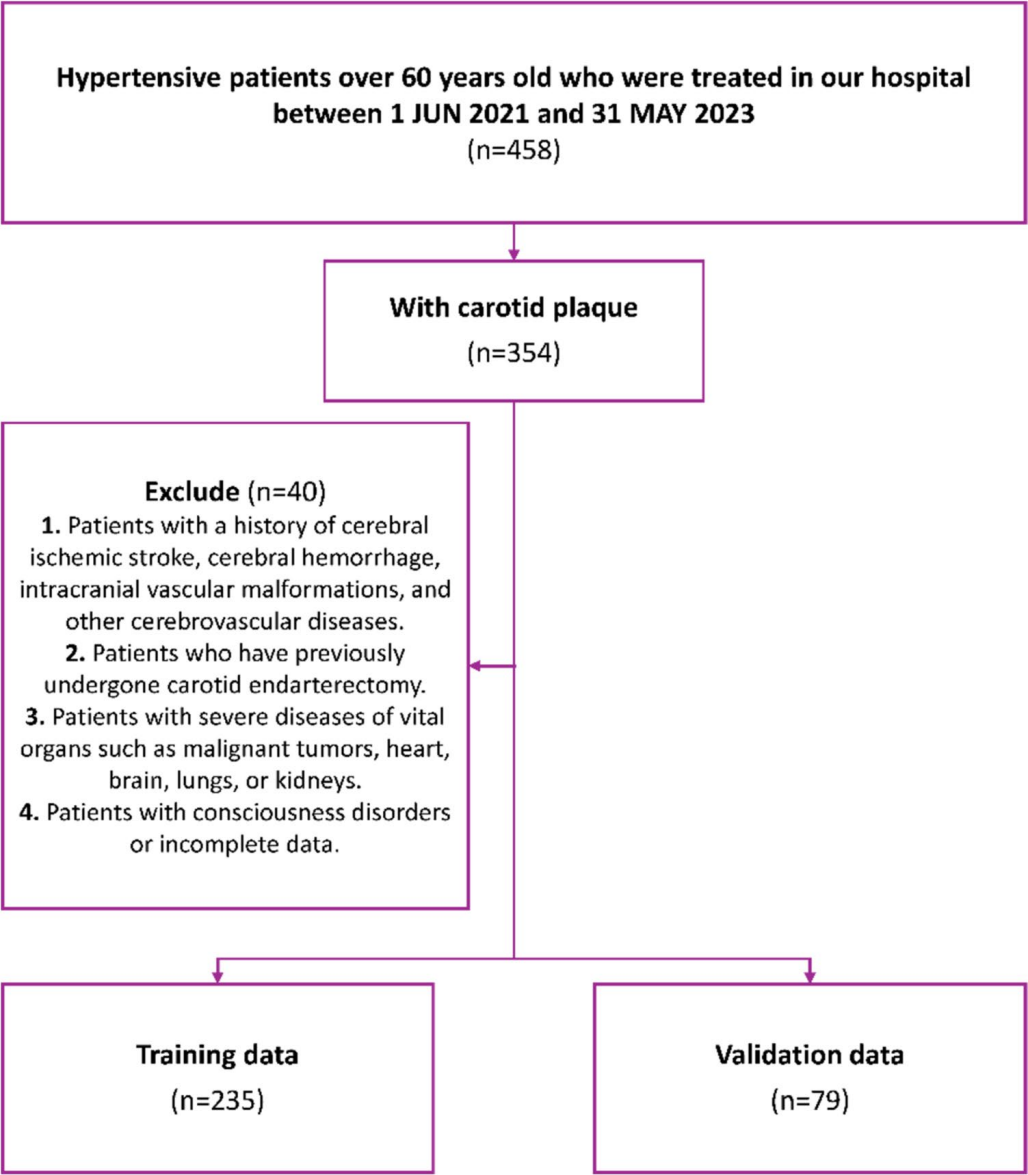
Flow chart showing selection of cohort

Based on the predefined inclusion and exclusion criteria, this study retrospectively included 314 elderly hypertensive patients with carotid plaques who were treated at our hospital between June 1, 2021, and May 31, 2023. The patients were randomly divided into a training cohort (235 patients) and a validation cohort (79 patients). They were categorized into a stroke group and a non-stroke group based on the diagnosis of ischemic stroke events (see Fig. 1).

### Inclusion and exclusion criteria

This study was reviewed and approved by the Ethics Committee of the Chengde Central Hospital (CDCHLL2023-469). All enrolled patients signed written informed consent forms, in accordance with the Declaration of Helsinki.

Inclusion Criteria: (1) Age ≥ 60 years; (2) Diagnosed with primary hypertension, according to 2018 ESC/ESH Guidelines for the management of arterial hypertension [8]. (3) Diagnosed with carotid plaque by SMI examination at our hospital; (4) The patient or their legal guardian signed the informed consent form.

Exclusion Criteria: (1) Patients with combined or existing brain space-occupying lesions, cerebral hemorrhage, intracranial vascular malformations, or a history of cerebrovascular disease; (2) Patients who have previously undergone carotid endarterectomy (CEA) or carotid artery stenting (CAS); (3) Patients with severe diseases of vital organs such as malignant tumors, heart, brain, lung, or kidney; (4) Patients with chronic inflammatory diseases, infectious diseases, or wasting diseases; (5) Patients with consciousness disorders or incomplete data.

### Criteria for hypertension diagnosis

According to the 2018 ESC/ESH Guidelines for the Management of Arterial Hypertension [8], hypertension is diagnosed when office blood pressure (BP) is ≥ 140/90 mmHg on at least two different occasions during separate visits, or if ambulatory BP monitoring shows a 24-h average BP of ≥ 130/80 mmHg, a daytime (awake) average BP of ≥ 135/85 mmHg, or a nighttime (asleep) average BP of ≥ 120/70 mmHg.

Hypertension is graded as follows: Grade 1 Hypertension is 140–159/90–99 mmHg, Grade 2 Hypertension is 160–179/100–109 mmHg, and Grade 3 Hypertension is ≥ 180/110 mmHg.

### SMI examination

For SMI examination, a TOSHIBA Aplio300 color Doppler ultrasound diagnostic instrument with a probe frequency of 3.0 to 11.0 MHz (model: 11L3) and built-in SMI imaging software was used. During the examination, the patient lay supine without a pillow, maintaining calm breathing, and the head was tilted back slightly toward the opposite side to fully expose the head and neck. Examination procedure: The target for observation was a plaque with a thickness of ≥ 2 mm and predominantly hypoechoic or mixed echogenicity. After clearly displaying the plaque image using two-dimensional ultrasound and keeping the probe in position, the patient was instructed to breathe calmly. The SMI mode was activated for examination, instrument parameters were set, and depth and gain were appropriately adjusted. The plaque was observed for more than 30 s to check for enhanced echoes within the plaque. Images were recorded and stored. SMI Detection Criteria for Neovascularization: continuous blood flow signals were detected in both transverse and longitudinal sections of the plaque. Transient or unstable flickering signals were considered artifacts and excluded.

SMI blood flow grading is classified into four levels [15]: grade 0, no blood flow signal within the plaque; grade 1, one or several punctate blood flow enhancement signals within the plaque; grade 2, punctate or 1–3 linear blood flow enhancement signals within the plaque; and grade 3, multiple linear enhancement signals within the plaque, some traversing the entire plaque (Supplementary Fig. 1).

During the SMI examination in this study, measurements and recordings were performed by the same doctor with over three years of experience in vascular ultrasound examination, following standard operating procedures.

### Clinical variables

Baseline characteristics: Gender, age, Body Mass Index (BMI), smoking history, and comorbidities (diabetes, hyperlipidemia, hyperuricemia, hyperhomocysteinemia, coronary heart disease, heart failure, COPD, gastritis and gastric ulcer, blood pressure system diseases) (Table 1).

**Table 1:** Baseline characteristics of hypertensive patients with carotid plaques

Characteristics	No ischemic stroke (n = 156)	With ischemic stroke (n = 79)	*P* value*
Age (mean (SD))	75.20 (6.67)	75.49 (5.72)	0.743
Gender (%)			
Male	76 (48.7)	39 (49.4)	0.991
Female	80 (51.3)	40 (50.6)	
Hypertension grade (%)			
Grade 1	42 (26.9)	12 (15.2)	0.017
Grade 2	65 (41.7)	28 (35.4)	
Grade 3	49 (31.4)	39 (49.4)	
BMI (kg/m^2^)	22.84 (4.13)	23.96 (3.73)	0.043
Smoking history (%)	91 (58.3)	49 (62.0)	0.686
Hyperlipidemia (%)	110 (70.5)	58 (73.4)	0.754
Diabetes (%)	80 (51.3)	46 (58.2)	0.384
Hyperuricemia (%)	62 (39.7)	40 (50.6)	0.147
Homocysteine (%)	55 (35.3)	20 (25.3)	0.163
Coronary disease (%)	44 (28.2)	22 (27.8)	0.989
Heart failure (%)	24 (15.4)	9 (11.4)	0.526
COPD (%)	18 (11.5)	6 (7.6)	0.475
Digestive ulcer (%)	8 (5.1)	9 (11.4)	0.138
Blood disorder (%)	5 (3.2)	3 (3.8)	0.998

*SD* standard deviation, *BMI* body mass index, *COPD* chronic obstructive pulmonary disease

Measurement data is expressed as mean and standard deviation (M(SD)); Count data is expressed as frequency and percentage (n (%))

*ANOVA for measurement data, exact *χ*^2^ for count data. *P* < 0.05 is considered statistically significant

SMI features: Plaque acoustic features, plaque length, plaque thickness, plaque surface morphology, percentage area of stenosis, intraplaque neovascularization (IPN) grade, and blood flow rate within the plaque microvasculature (flow rate) (Table 2).

**Table 2:** SMI characteristics of hypertensive patients with carotid plaques

Characteristics	No ischemic stroke (n = 156)	With ischemic stroke (n = 79)	*P* value*
IPN grade (%)			
Grade 0	59 (37.8)	13 (16.5)	0.013
Grade 1	46 (29.5)	25 (31.6)	
Grade 2	25 (16.0)	25 (31.6)	
Grade 3	26 (16.7)	16 (20.3)	
Flow rate (cm/s)	29.45 (4.73)	30.06 (5.13)	0.366
Plaque features (%)			
Homogeneous echo	8 (5.1)	8 (10.1)	0.375
Heterogeneous echo	27 (17.3)	10 (12.7)	
Low echo	36 (23.1)	16 (20.3)	
Isoechoic	23 (14.7)	14 (17.7)	
Mixed echo	28 (17.9)	13 (16.5)	
High echo	12 (7.7)	11 (13.9)	
Strong echo	22 (14.1)	7 (8.9)	
Plaque length (mm)	19.07 (13.25)	25.66 (12.48)	0.051
Plaque thickness (mm)	5.17 (3.38)	5.77 (3.51)	0.204
Surface morphology (%)			
Smooth	21 (13.5)	12 (15.2)	0.935
Ulcerative	84 (53.8)	42 (53.2)	
Movable	51 (32.7)	25 (31.6)	
Stenosis percentage (%)			
< 50%	52 (33.3)	5 (6.3)	0.011
50% ~ 70%	68 (43.6)	32 (40.5)	
> 70%	36 (23.1)	42 (53.2)	

*SD* standard deviation, *IPN* intraplaque neovascularization

Measurement data is expressed as mean and standard deviation (M(SD)); Count data is expressed as frequency and percentage (n (%))

*ANOVA for measurement data, exact *χ*^2^ for count data. *P* < 0.05 is considered statistically significant

### Statistical analysis

Statistical analysis was performed using R (version 4.4.0). Categorical data were described as percentages (%) or composition ratios, and group comparisons were performed using the *χ*^2^ test. Normally distributed measurement data were described as mean ± standard deviation (M ± SD), and group comparisons were performed using the independent samples *t* test. Student’s *t* tests, Wilcoxon rank-sum tests, and univariate logistic regression were used to select features. Finally, Least Absolute Shrinkage and Selection Operator (LASSO) regression analysis was employed to identify significant variables and construct a multivariate binary logistic regression prediction model. The validation group externally validated the prediction model using receiver operating characteristic (ROC) curves and calibration curves. A *P* value of less than 0.05 was considered statistically significant.

## Results

### Comparison of baseline characteristics between elderly hypertensive patients with carotid plaques in the ischemic stroke group and the non-stroke group

The training cohort included a total of 235 elderly hypertensive patients with carotid plaques who met the inclusion and exclusion criteria and underwent SMI examination. Among them, 79 patients (33.6%) were diagnosed with ischemic stroke, while the remaining 156 patients (66.4%) did not develop stroke events. Compared to the non-stroke group, the stroke group had significantly higher hypertension grades (*P* = 0.017) and higher BMI (*P* = 0.043), while there were no significant differences in gender, age, smoking history, and comorbidities between the two groups (*P* > 0.05) See Table 1.

### Comparison of SMI characteristics between elderly hypertensive patients with carotid plaques in the ischemic stroke group and the non-stroke group

Compared to the non-stroke group, the stroke group had higher IPN grades (*P* = 0.013) and a larger percentage area of stenosis (*P* = 0.011). Other SMI characteristics, including blood flow rate within the neovascularization of the plaque, plaque acoustic features, plaque surface morphology, plaque thickness, and plaque length, did not show significant statistical differences See Table 2.

### Univariate binary logistic regression analysis based on SMI and baseline parameters

**Fig. 2:**
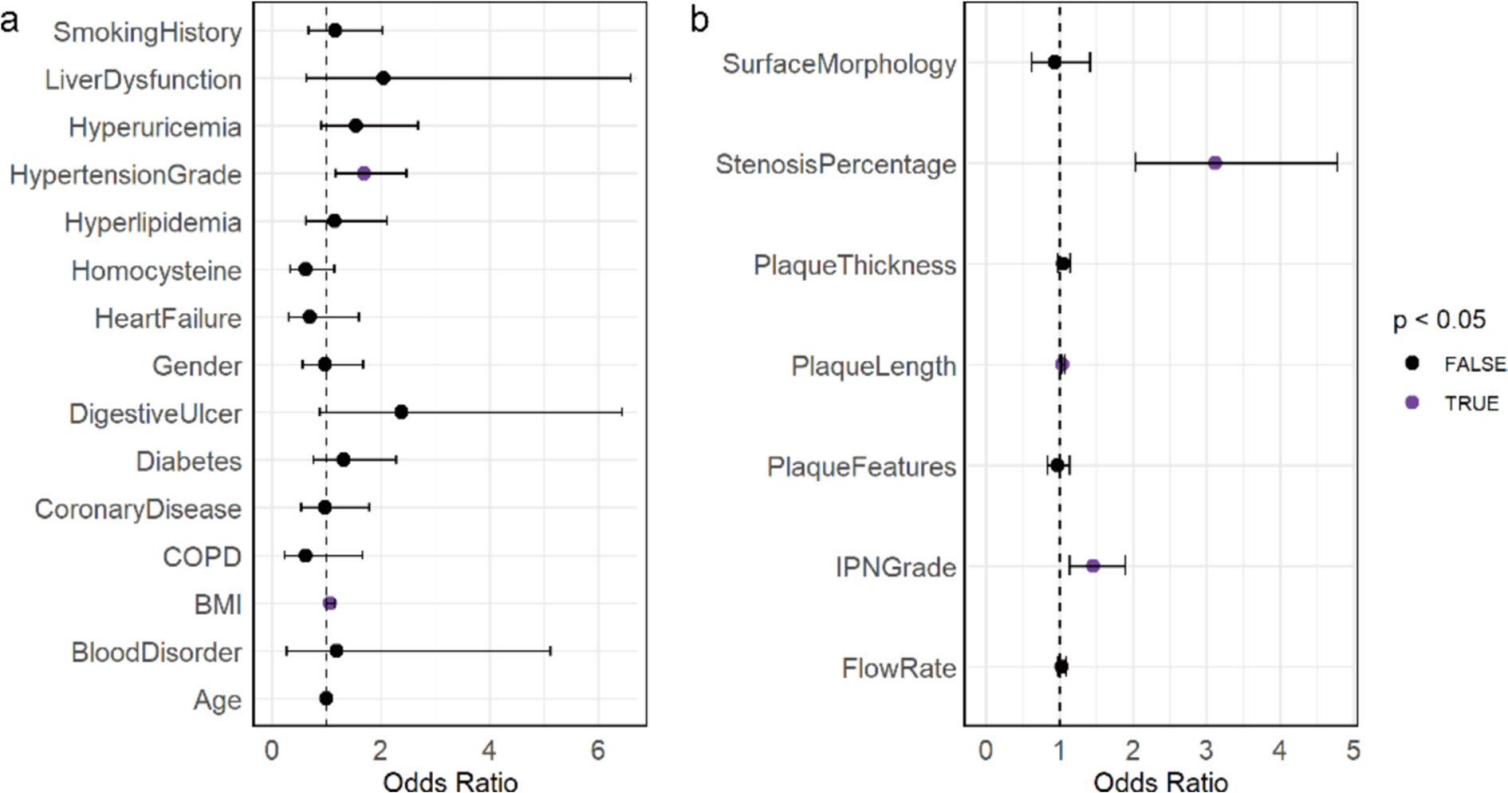
Forest plot of single variable logistic regression analyses. (**a**) Baseline features in predicting the risk of ischemic cerebrovascular events. (**b**) SMI features in predicting the risk of ischemic cerebrovascular events. Variables with *P* values < 0.05 are marked in purple, while variables with *P* values > 0.05 are marked in black

To further assess the association between the outcome and each SMI or baseline characteristics, we performed univariate logistic regression analysis, treating each characteristic as an independent variable. The results indicated that the regression coefficients for BMI and hypertension grade among the baseline characteristics, and IPN grade, plaque thickness, and stenosis percentage among the SMI characteristics, were statistically significant in the Wald test (*P* < 0.05), suggesting a significant correlation with the outcome events (Fig. 2, Table 3). Specifically, BMI (OR = 1.7), hypertension grade (OR = 1.07), IPN grade (OR = 1.80), plaque thickness (OR = 1.13), and stenosis percentage (OR = 2.72) were significantly positively correlated with an increased risk of ischemic stroke. This indicates that higher BMI, hypertension grade, and IPN grade, thicker plaques, and higher stenosis percentage all suggest an increased risk of developing ischemic stroke in elderly hypertensive patients (Fig. 2, Table 3).

**Table 3:** Univariate regression analyses for association between ischemic cerebrovascular event and SMI/baseline features

	Odds ratio	Lower 95% CI	Upper 95% CI	*P* Value*
SMI features				
IPN grade	1.80	1.37	2.35	0.002
Flow rate	0.97	0.91	1.02	0.260
Plaque features	1.09	0.93	1.27	0.308
Plaque length	1.11	1.05	1.17	0.018
Plaque thickness	1.13	1.00	1.28	0.041
Surface morphology	1.09	0.75	1.58	0.663
Stenosis percentage	2.72	1.81	4.08	0.002
Baseline features				
Age	1.01	0.97	1.05	0.742
Gender	0.97	0.57	1.67	0.925
Hypertension grade	1.70	1.17	2.47	0.005
BMI	1.07	1.00	1.15	0.045
Smoking history	1.17	0.67	2.03	0.586
Hyperlipidemia	1.15	0.63	2.12	0.641
Diabetes	1.32	0.77	2.29	0.314
Hyperuricemia	1.56	0.90	2.68	0.113
Hyperhomocysteinemia	0.62	0.34	1.14	0.124
Coronary disease	0.98	0.54	1.80	0.954
Heart failure	0.71	0.31	1.60	0.407
COPD	0.63	0.24	1.66	0.349
Digestive ulcer	2.38	0.88	6.43	0.088
Blood disorder	1.19	0.28	5.12	0.813
Liver dysfunction	2.05	0.64	6.59	0.226

*SD* standard deviation, *BMI* body mass index, *COPD* chronic obstructive pulmonary disease; *IPN* intraplaque neovascularization.

*P < 0.05 is considered statistically significant

### Variable selection using LASSO regression and construction of univariate and multivariate logistic regression prediction models

To improve the accuracy of the model and prevent overfitting, we employed the Least Absolute Shrinkage and Selection Operator (LASSO) regression analysis to further screen variables that were statistically significant in descriptive statistics and univariate analysis. These variables included BMI, hypertension grade, IPN grade, plaque thickness, and stenosis percentage. The optimal *λ* value was determined through cross-validation (Supplementary Fig. 2), and the final variables included in the equation were BMI, hypertension grade, IPN grade, and stenosis percentage.

**Fig. 3:**
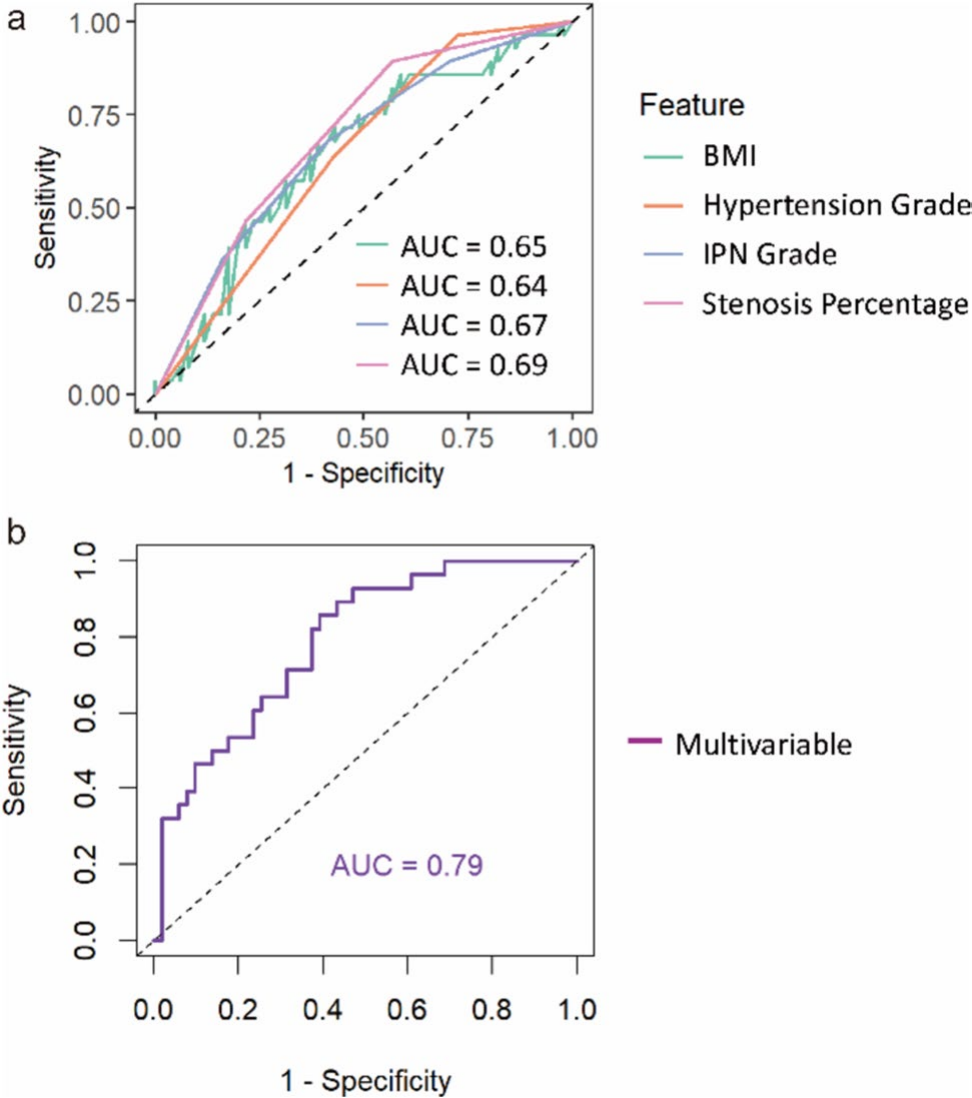
Receiver operating characteristic (ROC) curve for prediction of ischemic stroke incidence. (**a**) ROC curve for single variable logistic regression analyses. (**b**) ROC curve for multivariable logistic regression analysis

Based on these four selected factors, we used the receiver operating characteristic (ROC) curve to validate the predictive performance of each univariate logistic regression model. The results indicated that the predictive performance of the univariate logistic models was generally moderate. The area under the curve (AUC) for each univariate model was as follows: BMI 0.65, Hypertension grade 0.64, IPN grade 0.67, and Stenosis percentage 0.69 (Fig. 3**a**).

Next, we constructed a multivariate logistic regression prediction model combining the 4 selected variables. The likelihood ratio (LR) for this regression model was 42.6, with *P* < 0.001, indicating that the inclusion of multiple variables significantly improved the model's fit. The Cox & Snell R^2^ was 46.6%, indicating a good explanation of data variability. The regression prediction model equation is as follows:

$$\log (P/1 - P) = - 0.8487 + 0.2621 \times {\text{A}} + 0.2474 \times {\text{B}} + 0.3123 \times {\text{C}} + 07894 \times {\text{D}}$$

A, Hypertension Grade; B, BMI; C, IPN Grade; D, Stenosis Percentage.

### Validation of the multivariate prediction model in the cohort

To test the efficacy of this multivariate binary logistic regression model, we used the ROC curve to validate the model’s predictive performance in a validation cohort of 79 patients. The results showed that the model had an area under the curve (AUC) of 0.79 (95% CI: 0.69–0.89). At the optimal threshold, the prediction model's accuracy was 69.6%, sensitivity was 60.8%, and specificity was 85.7%, indicating much better predictive performance comparing to univariate models for predicting ischemic stroke events in elderly hypertensive patients (Fig. 3b).

## Discussion

In this study, we evaluated carotid plaque neovascularization and other acoustic features measured by SMI, and their association with ischemic stroke in elderly hypertensive patients. The primary findings indicate that higher BMI, hypertension grade, IPN grade, and the degree of carotid plaque stenosis are significantly associated with the occurrence of ischemic stroke. The LASSO-logistic regression model, incorporating these four variables, demonstrated a robust predictive ability with an AUC of 0.79, an accuracy of 69.6%, a sensitivity of 60.8%, and a specificity of 85.7%.

Currently, contrast-enhanced ultrasound (CEUS) is the most widely used examination method for effectively identifying early atherosclerotic plaques [9]. Studies have found that SMI technology can detect carotid plaque neovascularization with accuracy comparable to CEUS, offering a promising noninvasive alternative for assessing plaque stability [5]. Moreover, SMI has the advantages of convenience, non-invasiveness, and cost-effectiveness for clinical practice. This study again demonstrated the feasibility of SMI technology in identifying “vulnerable carotid plaque” that was significantly associated with ischemic strokes.

In previous studies, the relationship between intraplaque neovascularization and plaque instability has been well-documented, which subsequently contributes to plaque rupture and cerebrovascular events. Cui et al. [10], demonstrated that IPN, measured by contrast-enhanced ultrasonography (CEUS), is an independent predictor of future vascular events in patients with mild and moderate carotid stenosis. Accordingly, Huang et al. [11], showed that carotid plaque enhancement on contrast-enhanced ultrasonography is a significant and independent predictor of stroke recurrence in patients with ischemic stroke. In addition, BMI [12], hypertension grade [13], and degree of carotid artery stenosis [14] are known risk factors for ischemic cerebrovascular events. Our findings align with the earlier research that identified carotid plaque IPN as a critical factor in the pathogenesis of ischemic strokes.

Interestingly, other commonly acknowledged risk factors, such as age, smoking, hyperlipidemia, hyperuricemia, hyperhomocysteinemia, and diabetes showed no difference in predicting ischemic stroke. This may be due to differences in the selected cohort. Previous studies were usually based on the general population, while this study focused on hypertensive patients with carotid plaques.

## Strengths and limitations

The study utilizes a novel imaging technique, SMI, providing a non-invasive method for assessing carotid plaque vulnerability. A robust sample size and the inclusion of a validation cohort enhance the reliability and external validity of the predictive model. The use of LASSO regression ensures the selection of the most relevant variables, minimizing the risk of overfitting.

While the study highlights a significant association between the identified variables and ischemic stroke risk, the limitations should also be considered. First of all, the predictive model, although robust, might be influenced by unmeasured confounding factors such as genetic predisposition, medication adherence, and lifestyle factors not accounted for in the study. Additionally, the retrospective nature of the study may introduce selection bias, and **t**he study population is limited to elderly hypertensive patients, which potentially limited the applicability of the findings to other demographic groups. Last, this study lacks long-term follow-up data to assess the predictive model’s performance over extended periods.

Future studies may focus on prospective cohort designs to validate the predictive model in diverse populations and over longer follow-up periods. Additionally, incorporating genetic, behavioral, and other potentially influential variables could provide a more comprehensive understanding of ischemic stroke risk. Exploring the integration of SMI with other imaging modalities might also enhance diagnostic accuracy and risk stratification.

## Conclusion

In conclusion, this study identified four ischemic stroke risk-related indicators for elderly hypertensive patients with carotid plaques: BMI, hypertension grade, IPN grade, and carotid stenosis percentage. The logistic regression prediction model constructed based on these indicators can improve the accuracy and sensitivity of predicting ischemic stroke in elderly hypertensive patients. This provides a reliable predictive tool for clinical practice, helping to enhance the accuracy of clinical decision-making and improve patient prognosis.

## Supplementary Information

Below is the link to the electronic supplementary material.

Supplementary file1 (DOCX 156 KB)

## Data Availability

The data that support the findings of this study are available on request from the corresponding author upon reasonable request.
