# Supplementary material for: Prediction of ischemic stroke in elderly hypertensive patients using carotid plaque superb microvascular imaging characteristics: a lasso-logistic regression model
Source: J Med Ultrason (2001). 2025 Mar 15;52(2):227–35. doi: 10.1007/s10396-024-01513-0 (PMC12018490; doi:10.1007/s10396-024-01513-0)
Supplement: Supplementary file 1 — Supplementary file1 (DOCX 156 KB) [file 10396_2024_1513_MOESM1_ESM.docx]

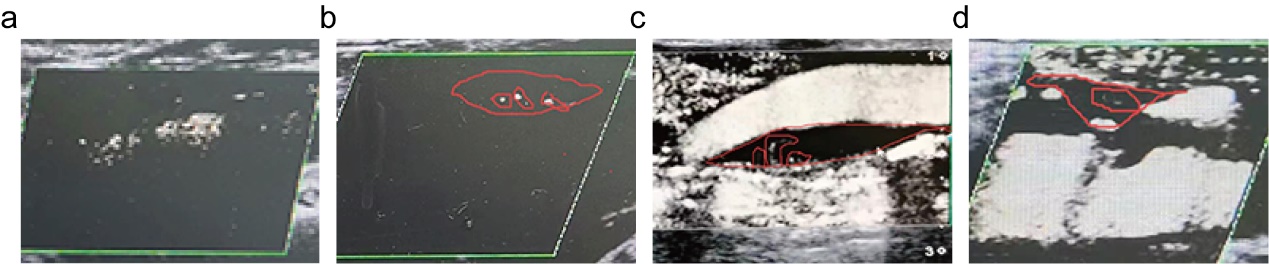


**Supplementary Figure 1. Representative images of carotid intraplaque neovascularization. a** IPN grade 0: no blood flow signal within the plaque. **b** IPN grade 1: one or several punctate blood flow enhancement signals within the plaque. **c** IPN grade 2: punctate or 1-3 linear blood flow enhancement signals within the plaque. **d** IPN grade 3: multiple linear enhancement signals within the plaque, some traversing the entire plaque.

**
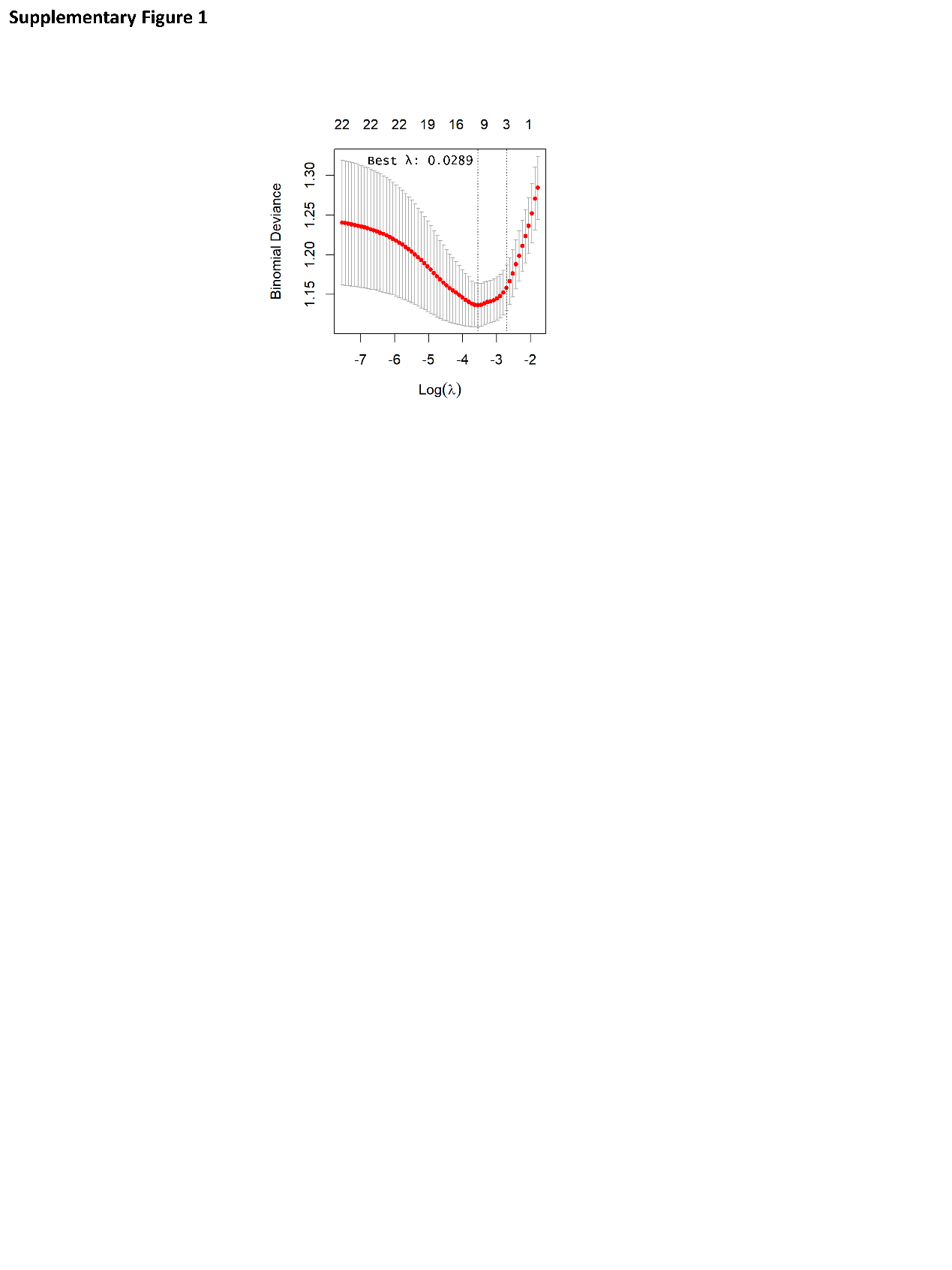
**

**Supplementary Figure 2. Cross-validation plot for LASSO regression.** Best lambda value is 0.0289, which minimizes the binomial deviance.
